# Bilateral Subthalamic Nucleus Deep Brain Stimulation under General Anesthesia: Literature Review and Single Center Experience

**DOI:** 10.3390/jcm9093044

**Published:** 2020-09-21

**Authors:** Hye Ran Park, Yong Hoon Lim, Eun Jin Song, Jae Meen Lee, Kawngwoo Park, Kwang Hyon Park, Woong-Woo Lee, Han-Joon Kim, Beomseok Jeon, Sun Ha Paek

**Affiliations:** 1 Department of Neurosurgery, Soonchunhyang University Seoul Hospital, Seoul 04401, Korea; c99867@schmc.ac.kr; 2 Department of Neurosurgery, Seoul National University Hospital, Seoul 03080, Korea; lim.yh@daum.net (Y.H.L.); moineun@daum.net (E.J.S.); 3 Department of Neurosurgery, Pusan National University Hospital, Busan 49241, Korea; geosung1@naver.com; 4 Department of Neurosurgery, Gachon University Gil Medical Center, Incheon 21565, Korea; medicwoo@gmail.com; 5 Department of Neurosurgery, Chuungnam National University Sejong Hospital, Sejong 30099, Korea; tooez4me@naver.com; 6 Department of Neurology, Nowon Eulji Medical Center, Eulji University, Seoul 01830, Korea; w2pooh@hanmail.net; 7 Department of Neurology, Seoul National University Hospital, Seoul 03080, Korea; movement@snu.ac.kr (H.-J.K.); brain@snu.ac.kr (B.J.)

**Keywords:** general anesthesia, intraoperative computed tomography, intraoperative magnetic resonance imaging, local anesthesia, microelectrode recording, Parkinson’s disease, subthalamic nucleus, deep brain stimulation

## Abstract

Bilateral subthalamic nucleus (STN) Deep brain stimulation (DBS) is a well-established treatment in patients with Parkinson’s disease (PD). Traditionally, STN DBS for PD is performed by using microelectrode recording (MER) and/or intraoperative macrostimulation under local anesthesia (LA). However, many patients cannot tolerate the long operation time under LA without medication. In addition, it cannot be even be performed on PD patients with poor physical and neurological condition. Recently, it has been reported that STN DBS under general anesthesia (GA) can be successfully performed due to the feasible MER under GA, as well as the technical advancement in direct targeting and intraoperative imaging. The authors reviewed the previously published literature on STN DBS under GA using intraoperative imaging and MER, focused on discussing the technique, clinical outcome, and the complication, as well as introducing our single-center experience. Based on the reports of previously published studies and ours, GA did not interfere with the MER signal from STN. STN DBS under GA without intraoperative stimulation shows similar or better clinical outcome without any additional complication compared to STN DBS under LA. Long-term follow-up with a large number of the patients would be necessary to validate the safety and efficacy of STN DBS under GA.

## 1. Introduction

Parkinson’s disease (PD) is the second most common neurodegenerative disease following Alzheimer’s disease, characterized by bradykinesia, rigidity, resting tremor and postural instability [1]. The long-term use of anti-Parkinsonian drugs has been found to be associated with dyskinesia and symptom fluctuation. Since the introduction of deep brain stimulation (DBS) in 1980s, DBS has been accepted as a preferred surgical treatment for PD [2]. Internal globus pallidus (GPi) and subthalamic nucleus (STN) are the main stimulation targets [3]. In particular, bilateral STN DBS is known to significantly improve not only primary motor symptoms, but also non-motor symptoms, such as sensory symptoms and sleep disturbances [4,5].

Traditionally, DBS surgery is performed under local anesthesia (LA) and conscious sedation to evaluate clinical benefit and side effects by localizing electrophysiological target using microelectrode recording (MER) and/or intraoperative test stimulation while the patient is awake [6,7,8,9,10,11,12,13,14,15]. STN DBS has several advantages when implemented under LA. The spike features of MER can be analyzed, and symptom relief or side effects by stimulation can be evaluated with intraoperative macrostimulation. In addition, by using electrophysiological targeting using MER, it is possible to compensate for errors from planning based on preoperative imaging, which is caused by brain shift due to cerebrospinal fluid (CSF) leakage after dura opening. However, MER under LA requires PD patients to withstand surgical procedure with approximately 18 h of antiparkinsonian medication discontinued. Most PD patients are old age and have severe multiple neuro-skeleto-muscular symptoms due to comorbidity, such as spinal stenosis and herniated intervertebral disc. Moreover, patients have to wear a frame on their head during the entire procedure and undergo surgery with the frame fixed to the operation table; thus, the patients may suffer from intolerable pain and psychological sequelae. The risk of hemorrhage risk also increases if an unintended large motion occurs due to cough or tremor during surgery. Patient cooperation is one of the factors that may influence the outcome after surgery.

Because of these concerns, many authors have consistently tried STN DBS under GA and reported that the clinical outcome is not inferior compared to under LA. However, there have been no randomized trials comparing DBS surgery under LA and GA due to logistical concerns. Only class II evidence has been compared through retrospective data analysis [16]. Here, we aimed to review previously published literature on STN DBS under GA as an alternative to STN DBS under LA. The technique and clinical outcome using intraoperative imaging and MER in DBS under GA are thoroughly reviewed along with the introduction of single-center experience of our institution.

## 2. STN DBS Using Intraoperative Imaging or Microelectrode Recording Under GA

The DBS surgical procedure can be divided into two stages: the intracranial implantation of DBS electrodes and the implantation of implantable pulse generator (IPG). In the case of IPG implantation, GA is generally preferred because tunneling is required subcutaneously. For intracranial electrode implantation, the STN DBS procedure under LA and GA are similar, but the specific details are different. The main difference between the STN DBS surgical procedure under LA and GA is the intraoperative verification method for the intended target acquisition, i.e., test stimulation or intraoperative imaging with or without MER. An accurate electrode location is a key factor to determine the postoperative prognosis after STN DBS surgery [17,18,19,20]. Image verification of the lead position is an important step, whether intra- or postoperatively [21]. For STN DBS under GA, some centers perform intraoperative verification using MER even under GA, and other centers use intraoperative imaging without MER. We reviewed each method of STN DBS under GA using intraoperative imaging or MER, respectively (Table 1).

### 2.1. Using Intraoperative Imaging

With the development of the quality of magnetic resonance imaging (MRI) over the past decades, it has become feasible to identify the STN boundary can to easily implement DBS under GA using direct targeting using advanced imaging [36]. The combination of direct targeting based on MRI visualization of anatomical structures and intraoperative imaging used to confirm accurate lead placement enables surgeons to accurately identify STN targets. It may allow STN DBS procedure to be performed in an asleep state under general anesthesia (GA) without neurophysiological test [37,38,39].

Successful clinical results on the intraoperative imaging to verify the accuracy of STN lead position instead of electrophysiological structure mapping or stimulation tests during DBS surgery have been reported [23,29,31,37,40,41,42,43,44]. In recent studies on the advancement of intraoperative imaging, no significant clinical results were found when compared to awake DBS [16,30,37,45,46]. However, most of these studies are retrospective analyses with a small number of patients and significant heterogeneity in anesthesia and surgical techniques. In addition, most of the studies were conducted in highly specialized centers with considerable experience in intraoperative imaging. Although these results may not be generalized to all DBS centers for these reasons, current results of STN DBS under GA are promising.

#### 2.1.1. Intraoperative CT

In some centers, intraoperative computed tomography (iCT) during surgery is used to verify the accuracy of lead placement (Table 1). This is achieved through fusion of iCT scans and preoperative MRI scans after intracranial electrode implantation [31,37,41,44,47]. In awake DBS surgery with MER guidance, iCT provides useful information, such as hemorrhage and a general idea of electrode location when fused with preoperative MRI [21]. According to a study about the accuracy of microelectrode trajectory in patients receiving MER-guided awake DBS using iCT, median (IQR) radial error 0.59 (0.64) mm, and median (IQR) absolute x and y coordinate errors were 0.29 (0.52) and 0.38 (0.44) mm, respectively [21]. Burchiel et al. fused and compared iCT and trajectory planning images after electrode implantation for various targets [37]. The mean vector error and mean deviation of trajectory was 1.59 ± 1.11 mm and 1.24 ± 0.87 mm, respectively, and the intraoperative replacement was performed on one electrode with a vector error of more than 3 mm. There was a significant correlation between the distance from the ventricle and the error. Kremer et al. stated that the mean difference between lead tips was 0.98 ± 0.49 mm, and the upper confidence interval did not exceed the non-inferiority margin described when comparing postoperative MRI with iCT [48].

Some centers use MER without test stimulation but with intraoperative imaging to verify the intended target acquisition in STN DBS surgery under GA [32,49,50,51,52,53,54,55,56,57,58]. A recent study compared the mean errors of MER-guided electrode implantation in DBS surgery under LA and those of iCT scan-guided intracranial electrode implantation in STN DBS surgery under GA [42]. When targeting STN, mean radial errors of the LA and GA group was about 0.9 ± 0.3 mm without significant difference (P = 0.70). The average number of brain penetration for electrode implantation in DBS surgery under LA and GA was similar (1.1 ± 0.2 and 1.1 ± 0.3 penetrations, *p* = 0.97). Brodsky et al. compared 6-months of the clinical outcomes between the group of LA and GA with iCT [59]. There was no significant difference in the improvement in UPDRS III and II, but the improvement in summary index (*p* = 0.004), subscores for cognition (*p* = 0.011), communication (*p* < 0.001), and speech outcome (category, *p* = 0.0012; phonemic fluency, *p* = 0.038) was found better in the GA group.

A few authors have published the results of a study using the intraoperative O-arm. Sharma et al. performed STN DBS surgery under GA using intraoperative O-arm without MER for various targets, and no significant targeting error due to incorporation of iCT images into preoperative CT or MRI was observed [60]. Carlson et al. also reported that intraoperative O-arm images provided a higher accuracy in determining the location of STN DBS electrodes than postoperative CT and MRI images [61].

#### 2.1.2. Intraoperative MRI

Other centers use intraoperative (interventional) MRI (iMRI) to guide DBS electrode placement to the STN (Table 1) [23,24,25,26,27,28,29,44,62,63,64]. For example, the UCSF group reported their experience about bilateral STN DBS in PD patients using a first-generation MRI system (Nexframe, high-field interventional MR-imaging) [25] and ClearPoint system (ClearPoint interventional MRI) [27]. There have been few published studies on the use of intraoperative MRI [23,24,26,27,28,44,62,63,64,65,66,67]. One of the reported advantages of iMRI is that it provides a real-time image acquisition to prospectively guided both trajectory planning and intended target verification prior to electrode placement [66]. Therefore, iMRI is one of the most useful methods for DBS targeting that allows precise validation of the real location of electrodes relative to the intended targets [66].

Researches using iMRI with or without stereotactic frame have shown that an accuracy of less than 1 mm can be achieved with mean error close to 0.7 ± 0.3 mm [22,23,25,27,64,66]. The main advantage of electrode implantation using iMRI is that electrode trajectory can be accurately implanted and adjusted before final placement by visualizing the intended target [66]. The error after correcting the electrode location using iMRI under GA without MER was similar to the error of using MER [30]. When comparing the electrode location on both sides, the error was smaller in the second insertion side than in the first insertion side, which is presumed to be due to the correction based on the iMRI result after the first insertion. Sidiropoulos et al. performed STN and GPi DBS surgery in advanced PD patients using the ClearPoint system and found that the mean radial error was 1.2 ± 0.7 mm in the STN group and 0.8 ± 0.3 mm in the GPi group [28]. Starr et al. et al. demonstrated a significantly lower rate of radial error compared to when inserted using the traditional frame-based stereotaxy (3.1 ± 1.41 mm) in the iMRI-guided placement group (1.2 ± 0.65 mm) through burr hole-mounted trajectory guide [22]. They explained that the possibility of brain shift-related errors was reduced because iMRI was performed after burr hole creation and intracranial air flow. Clinically, the UPDRS III “off” medication score and LEDD improved one year after surgery with iMRI [27].

#### 2.1.3. Targeting Accuracy

The theoretical assumption of STN DBS under GA surgery is that the accuracy in targeting STN is not less and the results are better than STN DBS surgery under LA using MER. Kochanski et al. analyzed MER trajectories after STN DBS using 227 iCTs and found that 1.2 ± 0.2mm of radial error occurred in comparison with the location of the intended targets [68]. These errors may be related to the mechanical errors related with the frame, arc, guide tube, and frame, which can lead to lead deviation [69]. In a large-scale study of DBS patients who underwent surgery using iCT, there were greater Euclidean error and greater medial deviation in the trajectory targeting Vim. The authors found that there are systematic tendencies in stereotactic error that differ with respect to the structure targeted [70]. In the study analyzing stereotactic accuracy of iMRI, the DBS lead placement using iMRI guidance showed a radial targeting error of 0.6–1.2 mm, while the error using iCT was 0.8–1.24 mm [22,25,27,28,31,37,71]. STN DBS surgery under GA using confirmatory iCT is based on the assumption that CT-MRI merge was performed correctly, but there may be some errors in the fusion of imaging modality, which may lead to suboptimal targeting [38,72,73]. The advantage of STN DBS surgery with iMRI guidance is that it has less dependence on image fusion and can reflect brain shift after dura opening. Analysis of the iMRI study revealed that the deep brain structure moves about 2 mm after opening the dura [74].

### 2.2. Using Microelectrode Recording

#### 2.2.1. Is MER Mandatory for STN DBS Surgery?

In the standard STN DBS procedure under LA, MER is used during surgery to obtain a signal to identify the deep structure [75]. The final site of electrode implantation is determined by considering both MER and intraoperative test stimulation [7,8,9,11,13,14,15]. Sedative drugs, such as propofol, dexmedetomidine, and remifentanil, are given to patients when it is not necessary for them to be awake [76,77]. The goal of using MER in STN DBS surgery is to obtain high accuracy in radiographic and neurophysiological targeting. Theoretically, the ideal target should be one and the same, but several important factors can lead to errors in targeting, resulting in inconsistency between optimal radiographic and neurophysiological targets. In the report on awake STN DBS, about 25% (38/150) of the electrodes were found very accurately located on the intended target very accurately with an error of less than 1mm, but electrophysiological recording did not match with the target in MER and/or intraoperative stimulation, or showed an unacceptably low side-effect threshold by stimulation [68]. Although these findings may be explained by brain shifts, these cases indicate that MER is essential for target confirmation during DBS surgery. Even small merge error combined with brain shift can lead to discrepancies between optimal radiographic and neurophysiological targets [38,72,74,78,79,80]. The advantage of this method is that it is possible to observe the changes in MER related to passive motion during surgery, and immediately evaluate the effects and side effects through test stimulation [9,81]. By reflecting this result and modifying the electrode position, the effect can be maximized while the complications of stimulation can be minimized.

MER signals may be mixed with many noises which may be caused by snoring or movements of the patient. The reliability and usefulness of MER during STN DBS surgery under LA are still being investigated. However, awake surgery may not be possible for some patients with severe anxiety, fear, reduced cooperation, severe pain, respiration difficulties and so on.

MER may increase the risk of intracranial hemorrhage and cognitive decline [82]. Binder et al. reported a bleeding rate of 3.3% and a risk of permanent defects 0.6% [83]. The number of MER trajectory was found slightly higher in patients with hemorrhage without statistical significance than the patients without hemorrhage [84]. Some researchers have also questioned whether MER has a real significant impact on target refinement [8]. They argued that a short MER-determined STN length alone cannot predict the occurrence of stimulation-related side effect [18]. Moreover, the MER procedure increases both surgical time and the cost [8,85].

Macrostimulation test cannot be performed if the patients are asleep during the operation. There is also controversy about whether intraoperative stimulation is needed during DBS surgery. Some researchers believe that it is necessary to confirm the effectiveness of the stimulus. On the other hand, some argued that discontinuation of the drug in LA makes the results less reliable, especially if it is not located in the correct position within the STN, the effect can be easily observed and difficult to distinguish from the lesion effect [86].

Due to the improved image quality of preoperative imaging, determining the final electrode location by imaging alone without MER does not negatively affect motor improvement and LEDD, and does not aggravate surgical complications [24,26,29,42,87]. The UPDRS III reduction rate at postoperative 3 months was higher in the group of STN DBS under LA with MER cohort (*p* = 0.006), but there was no significant difference at 1 year (*p* = 0.18), as well as in dysarthria, capsular, oculomotor, and sensory side effects [87]. Chen et al. also reported that there was no difference in the UPDRS III reduction rate and score 6 months after STN DBS surgery between the MER group and the non-MER group [42]. In addition to frequently used imaging sequences, direct targeting can be used with quantitative susceptibility mapping (QSM) and diffusion tensor imaging (DTI) [68].

#### 2.2.2. Is MER Possible Under GA?

STN DBS under GA has traditionally been used in patients who are unable to tolerate awake surgery including pediatric patients, or in patients who do not require clinical testing, such as obsessive-compulsive disorder or epilepsy. The biggest concern with STN DBS surgery under GA for movement disorder is the possibility of diminution of MER signals. A few small-sized retrospective studies have reported that MER obtained from STN, GPi, substantia nigra in STN DBS surgery under GA with both volatile and intravenous anesthetics in PD and dystonia patients showed no significant difference compared with patients awake during the procedure [54,88,89,90,91]. Notably, the neural activity of typical burst pattern disappeared when higher anesthetic doses were used. However, the results of these studies are controversial given the small sample size and heterogeneity of the anesthetic used. A prospective, double-blinded study is needed to compare the effects of anesthetic agents on MER quality in patients undergoing STN DBS surgery under GA.

The next concern is that since intraoperative stimulation cannot be performed under GA, immediate response of clinical effects and adverse effects associated with stimulation cannot be assessed during the STN DBS surgery. Several trials of MER in deep sedation have been performed without intraoperative stimulation [32,33,89]. In these studies, propofol or remifentanil tended to interfere with the electrophysiological signal, but there were no significant differences in terms of exact targeting, clinical effectiveness, and adverse event profiles. Other authors also reported that although there was significant MER signal attenuation in deep sedation with propofol, it did not interfere with the optimal approach to the target [32,33,92,93].

Although a few studies have previously investigated the effects of anesthetics on MER over the past 20 years, the exact effect has not been fully elucidated. Most studies were retrospective analyses with heterogeneity in the anesthesia protocol used and the patient population, and thus, no definitive conclusions could be drawn [77]. Therefore, most of the knowledge revealed to date is derived from the case reports or small case series. During MER, background neuronal discharges and spike activity patterns are an important part of the precise localization of the target nucleus. Anesthetics have been shown to affect background activity and neuronal spike activity in a dose-dependent manner, primarily through activation of γ-aminobutyric acid (GABA) receptors. In addition, anesthetics do not have the same effect on neuronal activity in various target nucleus. Since most anesthetics enhance the inhibitory action of GABA, this difference in GABA-input of the target nucleus plays an important role [94,95].

MER from STN in PD patients was successfully obtained under sedation with low-dose anesthetics. The anesthesia techniques used during MER ranged from conscious sedation with propofol, dexmedetomidine with no airway manipulation to GA with intravenous or inhalation anesthetics. Although anesthetics have been shown to reduce the spike activity, localization of the target areas was proven possible in most studies. Nevertheless, most studies did not mention the exact effect on the background activity, degree of suppression of spike activity, and the number of trajectories used for localization [34,58,80,89].

Under desflurane inhalation, Lin et al. observed that MER could be performed with a typical neuronal firing pattern and motion-related firing of STN, and the clinical results were similar in both groups [34,96].

Our group performed MER and implantation by administering propofol and fentanyl for sedation under LA, and reported the effects of propofol and fentanyl on MER and the clinical outcome. The locations of all electrodes were positioned within the STN. The postoperative 6-months UPDRS II and III, total “off” scores, Hoehn and Yahr (H&Y) scale, Schwab-England ADL scale scores, and LEDD have been greatly improved [92,93].

Although the effects of short-acting opioid receptor agonists, such as remifentanil, on MER are not well known, some data suggest that GABAergic neurons may play a central role [76,77,97]. A few reports showed that anesthesia using propofol reduces the firing rate of basal ganglia in a few reports [95,98], while one study showed no significant difference in firing rate compared to LA when administered with propofol and fentanyl [92]. Monitored anesthesia using propofol appears to be a safe technique for DBS procedure [99]. In some studies, MER was properly performed without affecting the surgical outcome only when remifentanil administration was discontinued and propofol was carefully monitored [32,54,100]. However, the spontaneous firing patterns of STN and substantia nigra remained similar to those under LA [14,100]. Chen et al. also reported that there was no significant difference between the GA and LA groups in terms of MER trajectory, recorded STN depths, postoperative coordinates, and overall incidence of stimulation-related side effect [55]. Under remifentanil or ketamine anesthesia, no significant differences were found in number of spikes detected, mean firing rate, pause index, and burst index compared to LA [57]. However, Moll et al. observed a long interburst between abnormally long group discharges under propofol and remifentanil [89].

Benzodiazepines are direct GABA-agonists, which can completely eliminate MER and cause dyskinesia. Dexmedetomidine may be a better alternative for anxiety relief. The effect of dexmedetomidine on neural activity has not been fully elucidated, but it seems to be a reasonable option due to the non-GABA-mediated mechanism of action. Several studies to date have shown minimal effects of low-dose dexmedetomidine on MER in STN and GPi [101,102,103,104]. Some authors reported that low doses of dexmedetomidine (<0.5 μg/kg/h) did not significantly affect the quality of MER in STN or GPi [76,99,103]. Although dexmedetomidine may affect the MER result, it does not affect target localization [50].

#### 2.2.3. Clinical Experiences of STN DBS Using MER under GA

Some authors performed STN DBS surgery on PD patients under GA and reported favorable clinical outcomes (Table 1). Hertel et al. reported that patients’ daily off phases decreased from 50% to 17%, while the Unified Parkinson’s Disease Rating Scale (UPDRS) III score was reduced from 43 (preoperative; medication off) to 19 (stimulation on; medication off) and 12 (stimulation on; medication on) [32]. Yamada et al. also reported that UPDRS II, III, IV on and off scores were significantly lower in the LA and GA groups at 3 months postoperatively, and the activities of daily living(ADL)s and motor symptoms, such as bradykinesia, tremor, rigidity, and axial symptoms, have improved significantly [54]. In this study, a reduction in dyskinesia duration (*p* < 0.001), disability (*p* = 0.009) and off period duration, and improvement of sleep disorders were observed. Other authors also reported significant improvement in off-medication UPDRS, levodopa-equivalent daily dose (LEDD), and quality of life [29,35]. Harries et al. reported a long-term clinical outcome of more than 5 years [49]. In their study, not only the UPDRS II and III off score, but also the total UPDRS off scores at postoperative 1 year improved significantly, and the total UPDRS score continued to improve for up to 7 years.

Previously, authors have suggested the use of bispectral analysis (BIS) of the electroencephalogram in STN DBS surgery under GA using MER. An appropriate MER signal can be easily obtained by adjusting the anesthesia depth using BIS [100,105]. BIS of 65–85 and 40–65 is recommended for sedation and GA, respectively [106]. In the case of sedation using dexmedetomidine, it has been reported that the MER signal does not differ from the nonsedated state if the BIS value is maintained below 80 [80].

### 2.3. Intraoperative Imaging vs. MER in STN DBS under GA

Recent meta-analysis reported that no significant difference was found in the improvement of UPDRS III score or LEDD between LA and GA cohort (Table 2, Table 3 and Table 4) [16,33,46,54,55,107]. Lefaucheur et al. reported that the rate of reduction in UPDRS III axial, gait, postural stability, and rigidity subscores tended to be greater when performed under LA compared to GA, but the difference was not statistically significant [33]. On the other hand, Chen et al. reported that the LA cohort showed greater improvement in posture and walking than the GA cohort (*p* = 0.054), while the GA cohort showed a significant decrease in cognitive function (*p* = 0.017) [55].

Some studies have used MER in STN DBS surgery under GA (mean 1.92 ± 0.68) and LA cohort (mean 2.27 ± 1.31) with respect to the maximum error of each read (*p* = 0.557) despite the varying targets [33,52,55]. Ho et al. reported that there was no significant difference between GA (mean 1.92 ± 0.68) and LA cohort (mean 2.27 ± 1.31) with respect to the maximum error of each lead (*p* = 0.557), but their study included a variety of targets [16]. The number of lead passes and the incidence of intracranial hemorrhage and infection were lower in STN DBS under GA, but treatment-related side effects based on the UPDRS IV “off” score were lower in DBS under LA (LA cohort 78.4% vs GA cohort 59.7%, *p* = 0.022) [16,35]. However, other studies showed no difference in the UPDRS IV subscore between the GA and LA groups [24,107]. As for LEDD, some studies reported that the 6-months postoperative LEDD reduction was significantly greater in the LA group, while others showed statistically similar reductions (LA cohort 38.27%, GA cohort 49.27%, *p* = 0.4447) [26,107]. Tsai et al. reported that symptoms of the patients with PD improved after DTN DBS in both LA and GA cohorts without significant differences in LEDD and UPDRS IV scores [52].

When the long-term outcome was investigated, the authors found that the probability of side effects by stimulation and lead revision was higher in the GA cohort without MER and test stimulation [68]. On the other hand, no difference was observed in UPDRS III score, LEDD, stimulation parameters, coordination of targeting, STN recording length, and side effects in the two groups [108].

STN DBS surgery can be safely performed with a low complication rate in both LA and GA cohort, and the results of the studies to date show that there is no significant difference in complication rates between the two groups. Some authors reported that overall DBS-related complications, such as intracranial hemorrhage (GA 0.3% vs LA 1.1%) and infection (GA 0.7% vs LA 1.4%), were significantly lower in GA cohort (*p* < 0.001) [16,35]. Martin et al. reported the incidence of hardware infection is due to electrode implantation after 10 years of MRI-guided STN DBS surgery [109]. In the study, the overall infection rate of 164 iMRI-guided surgeries with 272 electrodes implanted was 3.6%, which was similar to that reported in the previous STN DBS surgery under LA. The results of a systematic review on the incidence of complications, hospitalization time, and readmission rate of patients who underwent awake and asleep STN DBS surgery were recently published, and there was no statistical difference in the complication rate, length of hospitalization, and readmission rate of LA and GA cohort [110].

The mean total cost of STN DBS surgery under GA and LA was similar at $38,850 ± $4830 in GA and $40,052 ± $6604 in LA, respectively, but the standard deviation in DBS under GA was significantly lower [111]. This indicates that there is no difference in the total cost of DBS surgery under GA and LA, but the cost fluctuation is lower due to the lower incidence of unexpected variables in DBS surgery under GA. However, there are limitations to generalizing such result, since it is a single-center experience.

**Table 2:** Summary data of published literature comparing clinical outcome effect of after subthalamic nucleus deep brain stimulation under general anesthesia and local anesthesia in patients with Parkinson’s disease: Baseline patient characteristics

Author	Year	Study Type	Number of Patients		Age (yrs)		Disease Duration (yrs)		Follow-Up (Months)
			GA	LA	GA	LA	GA	LA	
Maltete et al. [58]	2004	Clinical	15	15	59.8 8.0	58.0 6.1	13.4 3.7	13.5 2.6	6
Yamada et al. [54]	2007	Clinical	15	10	65.2 7.0	65.6 8.6	11.1 5.0	6.8 2.4	3
Saleh et al. [26]	2015	Clinical	14	23	64.0 ± 11.9	60.6 ± 7.0	10.9 ± 3.8	11.3 ± 4.9	6
Tsai et al. [52]	2016	Clinical	8	8	49.6 ± 7.1 *	41.1 ± 10.2 *	9.3 ± 2.4	12.4 ± 9.2	6
Brodsky et al. [59]	2017	Clinical	27 (20 GPi, 7 STN)	34 (20 GPi, 14 STN)	63.7 ± 9.79	63.1 ± 7.61	NR	NR	6
Lefranc et al. [112]	2017	Clinical	13	10	62.80 ± 7.1	63.1 ± 10	12.60 ± 3.6	12.10 ± 3.5	12
Blasberg et al. [87]	2018	Clinical	48	48	65.75 ± 1.18	65.52 ± 1.13	11.65 ± 0.81	10.87 ± 0.78	6
Chen et al. [42]	2018	Clinical	41	14	64.6 ± 8.25	63.1 ± 10.1	7.5 ± 3.4	8.6 ± 4.6	6
Ho et al. [16]	2018	Meta-analysis	663	6441	58.3 ± 6.8	59.4 ± 5.2	11.0 ± 1.5	12.3 ± 2.1	12
Liu et al. [113]	2019	Meta-analysis	967	556	NR	NR	NR	NR	NR
Tsai et al. [108]	2019	Clinical	22	9	57.7 ± 7.4	49.4 ± 12.2	57.7 ± 7.4	49.4 ± 12.2	60
This study	2020	Clinical	90	56	57.43 ± 7.85	58.91 ± 8.65	11.67 ± 4.75	10.55 ± 4.89	6

Data are presented as: mean ± standard deviation GA, general anesthesia; LA, local anesthesia; GPi, Internal globus pallidus; STN, subthalamic nucleus; NR, Not reported * Age of Onset.

**Table 3:** Summary data of published literature comparing clinical outcome effect of after subthalamic nucleus deep brain stimulation under general anesthesia and local anesthesia in patients with Parkinson’s disease: Baseline and Follow-up Unified Parkinson’s Disease Rating Scale (UPDRS) III score and Levodopa equivalent daily dose (LEDD).

	Baseline UPDRS III		Follow-Up UPDRS III *		%UPDRS III Change			Baseline LEDD		Follow-Up LEDD		%LEDD reduction		
Author	GA	LA	GA	LA	GA	LA	*p* Value	GA	LA	GA	LA	GA	LA	*p* Value
Maltete et al.	47.1 ± 15.4	39.9 ± 13.9	17.0 ± 8.6	10.9 ± 7.2	63.9%	72.7%	0.07	1449 ± 398	1507 ± 465	310 ± 350	392 ± 440	78.6%	74.0%	0.06
Yamada et al.	52.4 ± 19.0	45.9 ± 17.7	14.3 ± 15.4	7.1 ± 7.0	72.7%	82.5%	No significant difference	375.7 ± 195.6	425.0 ± 171.8	303.3 ± 164.7	261.1 ± 164.0	16.6%	38.2%	NR
Saleh et al.	NR	NR	NR	NR	NR	NR	NR	2134.9 ± 1175.8	1702.7 ± 876.0	NR	NR	49.27%	38.27%	0.4447
Tsai et al.	41.7 ± 29.4	39.9 ± 16.3	NR	NR	65.7%	45.8 ± 26.2%	NR	NR	NR	NR	NR	NR	NR	NR
Brodsky et al.	42.2 ± 10.6	41.7 ± 12.5	14.8 ± 8.9 **	17.6 ± 12.26 **	35%%	42.2%%	0.19	NR	NR	NR	NR	NR	NR	NR
Lefranc et al.	35.92 ± 11.15	33.10 ± 5.38	18.0 ± 7.2	20.0 ± 10.47	49%	40.30%	0.336	1585.10 ± 496.40	1247.70 ± 579.80	519.17 ± 282.71	716.80 ± 320.14	Significantly greater in the GA than in the LA		0.03
Blasberg et al.	38.47 ± 1.94	34.79 ± 1.61	NR	NR	NR	NR	0.18	1070.72 ± 49.67	972.23 ± 55.15	NR	NR	NR	NR	0.008
Chen et al.	53.8 ± 16.4	53.7 ± 17.0	26.1 ± 12.0	21.6 ± 7.3	48.8%	40.3%	0.20	NR	NR	NR	NR	NR	NR	0.49
Ho et al.	NR	NR	NR	NR	51.1 ± 16.6% (n = 510)	46.7 ± 27.4 ± (n = 4931)	0.494	NR	NR	NR	NR	45 ± 12.8% (n = 444)	47 ± 26.6% (n = 3893)	0.752
Liu et al.	NR	NR	NR	NR	NR	NR	0.60	NR	NR	NR	NR	NR	NR	0.23
Tsai et al.	46.3 ± 14.4	28.6 ± 9.3	42.9 ± 17.4	24.6 ± 7.8	43.2 ± 14.1%	46.8 ± 13.8%	0.45	NR	NR	NR	NR	47.56 ± 18.98%	51.37 ± 31.73%	0.51
This study	38.11 ± 13.96	40.42 ± 15.30	21.48 ± 12.33	24.68 ± 12.51	43.6%	38.9%	0.136	1448.0 ± 546.93	1031.63 ± 451.08	483.99 ± 330.42	461.3 ± 284.65	66.6%	55.3%	<0.0001

Data are presented as: mean ± standard deviation. UPDRS, Unified Parkinson’s Disease Rating Scale; LEDD, Levodopa equivalent daily dose; GA, general anesthesia; LA, local anesthesia; GPi, Internal globus pallidus; STN, subthalamic nucleus; NR, Not reported * off medication, on stimulation ** recorded as reduced score.

**Table 4:** Summary data of published literature comparing clinical outcome effect of after subthalamic nucleus deep brain stimulation under general anesthesia and local anesthesia in patients with Parkinson’s disease: Perioperative complications.

Author	Number of MER Tracks	Overall Adverse Effects	Hemorrhage	Infection	Operation Time
Maltete et al.	NR	No adverse reaction to the use of propofol, 1 pulmonary atelectasia	NR	NR	NR
Yamada et al.	NR	NR	NR	NR	NR
Saleh et al.	NR	No significant differences	NR	NR	GA 424 ± 12 vs LA 307 ± 80 *p* = 0.0026
Tsai et al.	NR	No significant differences	NR	NR	NR
Brodsky et al.	NR	NR	1 small venous hemorrhage in LA, 1 small nonhemorrhagic infarct in GA	1 in GA	NR
Lefranc et al.	NR	No significant differences *p* = 0.39	NR	NR	NR
Blasberg et al.	NR	No significant differences	1.00	1.00	0.31
Chen et al.	NR	NR	NR	NR	GA 266.0 ± 60.6 vs LA 260.9 ± 57.6 *p* = 0.78
Ho et al.	GA 1.4 ± 0.44 vs LA 2.1 ± 0.69 *p* = 0.006	NR	%ICH/lead: GA 0.3 ± 0.0 vs LA 1.1 ± 0.3, *p* < 0.001	%infection/lead GA 0.7 ± 0.0 vs LA 1.4 ± 0.0, *p* < 0.001	GA 253.7 ± 82.3 vs LA 272.4 ± 92.5 *p* = 0.748
Liu et al.	NR	0.94	0.64	NR	0.47
Tsai et al.	Significantly less in GA *p* = 0.04	Similar adverse effects	NR	NR	NR
This study		1 required revision due to inappropriate lead position in LA		1 IPG site infection treated by antibiotics in LA	

MER, microelectrode recording; NR, not recorded; GA, general anesthesia; LA, local anesthesia; IPG, implantable pulse generator.

## 3. SNUH Experience

Our group have been implementing STN DBS under LA since 2005 initially under sedation using propofol and fentanyl from 2011 to 2014 [93], and under full GA since 2014. To determine if there is a difference in the clinical outcome of PD patients who received bilateral STN DBS under LA and GA, we compared the clinical outcomes of the consecutive 57 patients who received bilateral STN DBS under LA from 2005 to 2006 and consecutive 90 patients who received bilateral STN DBS under GA from 2014 to 2019. Because our group previously published a study on the clinical course and electrode location of patients who received bilateral STN DBS under LA [114,115], these patients were included for the comparison. After approval by the institutional review board (IRB No. 1904–015–102), we retrospectively reviewed all patient medical records and databases (unpublished data). The scales that evaluated patients were as follows: UPDRS, Hoehn and Yahr (H&Y) Staging, Schwab & England ADL, dyskinesia disability, LEDD, Short Form-36 Health Survey (SF-36), and neuropsychological tests. All clinical evaluations were performed before surgery and 6 months after surgery by experienced neurologists. Patients were evaluated in both off- and on-medication states, respectively.

STN DBS under general anesthesia was performed with maintenance of the BIS around 60-70, and MER was administered under general anesthesia. The characteristic discharges of the bilateral STN were identified using MER by LeadPoint (Medtronic, Minneapolis, MN). The permanent quadripolar electrodes were implanted along the proper trajectory to stimulate more sensorimotor region of the STN. The STNs were localized by a combination of brain MRI and intraoperative MER. We did not use an intraoperative macrostimulation technique [15]. The stereotactic frame was removed and the implantable pulse generators (IPG) (Medtronic, Minneapolis, MN, USA) were implanted in a subcutaneous pocket below both clavicles under general anesthesia in a single session. Electrical stimulation was started one day after surgery. The patients also took medications but at a reduced dose compared to their previous dose. The medications and stimulation parameters were progressively adjusted using an N′vision1programmer (Medtronic, Minneapolis, MN, USA) according to the clinical status of the patients.

**Figure 1:**
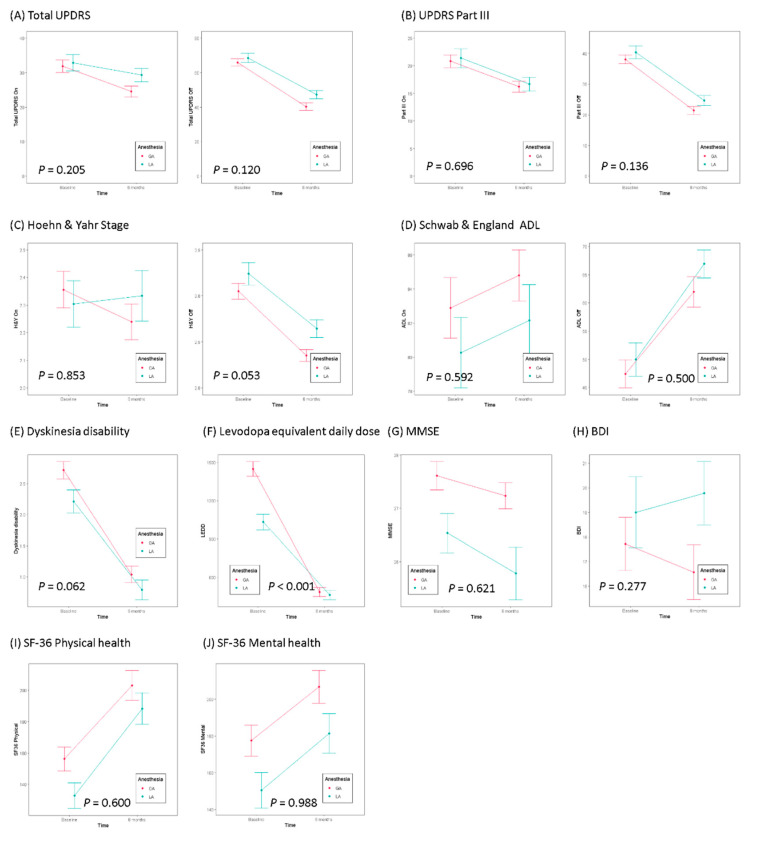
Comparison of clinical outcomes between baseline and 6 months after STN Deep brain stimulation (DBS) under local anesthesia (LA) and general anesthesia (GA) each cohort. (**A**) Total Unified Parkinson’s Disease Rating Scale (UPDRS) and (**B**) UPDRS part III showed significant improvement after 6 months compared to baseline, except for LA cohort medication on state, there was no statistically significant difference between LA and GA cohort. (**C**) Hoehn & Yahr stage and (**D**) Schwab & England activities of daily living (ADL) showed no significant change in the medication on state in both LA and GA cohort, and no significant difference between two cohorts. (**E**) Dyskinesia disability and (**F**) Levodopa equivalent daily dose (LEDD) were significantly decreased in both LA and GA cohort. Only LEDD showed a significant difference in the change between LA and GA cohort. (**G**) Mini Mental State Examination (MMSE) and (**H**) Beck’s Depression Inventory (BDI), showed no statistically significant decrease in both LA and GA cohort. (**I**) Short form -36 (SF-36) physical health and (**J**) Short form -36 (SF-36) mental health showed no statistically significant increase in both LA and GA cohort.

**Figure 2:**
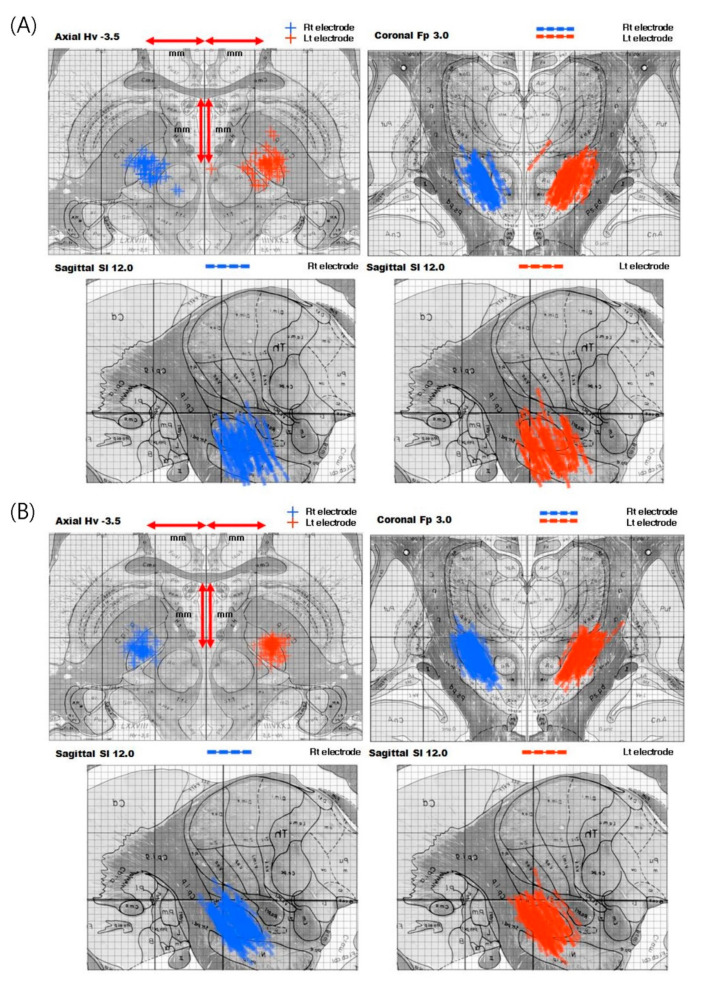
Plotting of the electrode location based on the plotted position of the electrode in the axial view which is 3.5 mm below the anterior commissure (AC)–posterior commissure (PC) line in the human brain atlas of Schaltenbrand and Wahren. (**A**) Local anesthesia (LA) cohort, (**B**) General anesthesia (GA) cohort. Compared to LA cohort, the GA cohort showed a higher tendency for the electrode to be located within the subthalamic nucleus (STN).

Statistical analyses were performed using SPSS software (SPSS statistics 18.0; SPSS Inc.). The data for the aforementioned variables were presented as the mean ± standard deviation using unpaired Student t tests. Mann–Whitney U-test and the Wilcoxon signed-ranked test were used for categorical data comparisons as appropriate. *p* values < 0.05 were considered statistically significant. Table 5 represents the patient characteristics and clinical scales of LA and GA cohort before DBS surgery. At baseline before surgery, the GA cohort showed higher LEDD, Beck’s Depression Inventory (BDI), and Short Form-36 (SF-36), and lower Beck Depression Inventory than the LA cohort. Table 6 shows the comparison between baseline and 6 months after DBS for each scale in LA and GA cohort. Total UPDRS and UPDRS III showed significant improvement after 6 months compared to baseline, except for LA cohort in on-medication state. H&Y stage and ADL score showed no significant change in the on-medication state in both GA and LA, and significantly decreased in the off-medication state. Dyskinesia disability and LEDD were significantly decreased in both GA and LA cohort. There was no significant change in Mini Mental State Examination (MMSE) and BDI after surgery in both groups. Physical health measured by SF-36 increased in both LA and GA cohort, and mental health showed no statistically significant increase. When analyzing the difference between the LA and GA cohort in the baseline of each item and the change after 6 months, only LEDD showed a significant difference (*p* < 0.0001). As shown in Figure 1, the degree of reduction in LEDD was greater in the GA cohort than in the LA cohort. We plotted the electrode location in each group based on the plotted position of the electrode in the axial view which is 3.5 mm below the anterior commissure(AC)-posterior commissure(PC) line in the human brain atlas of Schaltenbrand and Wahren (Figure 2). The electrode location on both sides in the LA group (n = 56) were as follows: both within STN (n = 30, 53.6%), only one within STN (n = 18, 32.1%), and both outside STN (n = 8, 14.3%). It was as follow in the GA group (n = 90): both within STN (n = 69, 76.7%), only one within STN (n = 20, 22.2%), and both outside STN (n = 1, 1.1%). There was a significant difference in the electrode location on both sides between the two groups (*p* = 0.001). Compared to the LA cohort (Figure 2A), the GA cohort (Figure 2B) showed a higher tendency for the electrode to be located within the STN. However, it should be interpreted in consideration of the fact that our group performed DBS surgery under LA in the early days, and under GA after more experienced. As intra- or postoperative complications, one revision and one infection occurred in LA cohort. One patient required revision surgery after 2 months due to inappropriate lead location. The other patient had IPG site infection, which improved after antibiotics treatment. In our center, postoperative MRI was taken 1 month after electrode implantation, so we cannot find post-electrode edema (PEE) in most cases. As recent studies have revealed that PEE is not simply a complication due to venous congestion and has no significant relationship with the number of tracks, further studies on the occurrence pattern of PEE under GA would be required [116,117,118,119].

## 4. Future Direction

Studies published to date have shown that the rationale and technology of STN DBS surgery performed under GA are accurate, and they presented similar clinical results compared to STN DBS under LA cohort. A large-scale prospective randomized controlled trial is in progress to assess the degree of the improvement of non-motor symptoms in PD patients [120].

Care should be taken when interpreting and applying the conclusion, since the STN DBS surgery under GA data reported to date have been published in large centers with considerable experiences. In general, STN DBS surgery should be performed in the most convenient way for the surgeons and center to provide the best results to the patients. Traditionally, factors, such as claustrophobia, severe off-medication symptoms, or nonspecific fear of waking during surgery, made patients choose GA. However, based on the increasingly cumulative data showing similar or better results compared to LA, a surgeon may choose STN DBS surgery under GA.

Adaptive DBS is a promising technology because it can provide more selective stimulation trigger/parameter and reduce stimulation-induced dyskinesia by suppressing beta activity when it exceeds a certain threshold level [121,122]. There is still little literature on adaptive DBS implemented under general anesthesia, and further studies for application of adaptive DBS under general anesthesia should be conducted.

There are patients who cannot undergo STN DBS surgery due to various reasons or may not benefit from STN DBS surgery. Non-invasive lesion-based therapies, such as focused ultrasound and Gamma Knife radiosurgery (GKRS), have been proposed as alternatives to DBS because of their effectiveness and safety [123,124,125,126]. The further innovative refinement of noninvasive methods of Gamma Knife radiosurgery (GKRS) and focused ultrasound may allow advanced PD patients to receive surgical treatment more conveniently and efficiently in the near future.

## 5. Conclusions

The number of DBS surgeries continues to increase, as indications expand and the population is aging. Currently, STN DBS surgery is performed in various ways with or without MER under LA or GA in each center. Based on the reports of previously published studies and ours, it is likely that GA does not interfere with the MER signal from STN. In addition, STN DBS under GA without intraoperative stimulation shows similar or better clinical outcome without any additional complication compared to STN DBS under LA. Although there are various pros and cons of each method in each protocol in each protocol of STN DBS under LA and under GA, the stereotype that STN DBS surgery must be performed under LA to perform intraoperative macrostimulation and MER to obtain the best clinical outcome should be changed at the moment.

In conclusion, it is suggested that, if there is no significant difference in clinical treatment effects and complications between GA and LA, it would be reasonable to implement STN DBS under GA because it can minimize unnecessary inconvenience of the patients with PD. Long-term follow-up studies with the large number of the patients would be necessary to further validate the safety and efficacy of STN DBS under GA.

## Figures and Tables

**Table 1:** Summary data of published literature presenting clinical outcome effect of after subthalamic nucleus deep brain stimulation under general anesthesia in patients with Parkinson’s disease.

Author	Year	No. of Patients	Age (yrs)	Disease Duration (yrs)	Follow-Up Months	UPDRS III Medication Off				LEDD			
						Baseline	Follow-Up *	% Change	*p*-Value	Baseline	Follow-Up *	% Change	*p*-Value
Intraoperative imaging													
Interventional MRI													
Starr et al. [22]	2010	29	58 ± 8.1	NR	9	49 ± 13	19 ±14	60%	0.0001	NR	NR	NR	NR
Foltynie et al. [23]	2011	79	58.9± 7.7	11.5 ± 7	14	51.5 ± 14.9	23.8 ± 11.2	52%	0.0001	NR	NR	NR	NR
Nakajima et al. [24]	2011	14	56.1 ± 6.5	13.8 ± 8.1	12 ± 6.1	57.9 ± 16.6	27.3 ± 11.8	53%	0.0001	1505 ± 764	764 ± 435	49.20%	<0.01
Ostrem et al. [25]	2013	17	59.8	11.1	6	44.5 ± NA	22.5	49.44%	0.001	1337 ± 482	NR	24.70%	0.003
Saleh et al. [26]	2015	14	64 ± 11.9	10.9 ± 3.8	5.86 ± 1.15	NR	NR	NR	NR	NR	NR	49.27%	0.0031
Ostrem et al. [27]	2016	20	63.2 ± 6.8	10.8 ± 2.9	12	40.75 ± 10.9	24.35 ± 8.8	40.20%	0.001	1072.5 ± 382	828.25 ± 492	21.13%	0.046
Sidiropoulous et al. [28]	2016	12	64.7 ± 5.9	11.9 ± 3.7	13.5 ± 3.7	37.2	20.2	46.2%	0.03	1458 ± 653	1337 ± 733	8.3%	0.7
Chircop et al. [29]	2018	26	60.2 ± 9.3	8.8 ± 2.7	12	45.9 ± 14.3	26.7 ± 11.5	41.70%	<0.001	863 ± 211	599 ± 273	30.60%	<0.001
Matias et al. [30]	2018	33	67.2 ± 6.4	12.7 ± 6.9	9.1	52.8 ± 14.9	28.6 ± 11.9	45.8%	<0.001	NR	NR	NR	NR
Intraoperative CT													
Mirzadeh et al. [31]	2016	35	61.1	10.7	6	48.4 ± 13.8	28.9 ± 12.5	40.30%	<0.0001	1207 ± 733	1035 ± 478	14%	0.004
Microelectrode recording													
Hertel et al. [32]	2006	9	70.7 ± 3.6	13.6 ± 6.0	3	43.0 ± NR	19 ± NR	55.80%	NR	NR	NR	NR	NR
Lefaucheur et al. [33]	2008	30	57.7 ± 11.1	14.0 ± 4.0	12	47.9 ± 13.6	48.6 ± 19.0	69.10%	0.87	1470.8 ± 729.5	NR	66.4 ± 17.2	NR
Lin et al. [34]	2008	10	58.9 ± 9.9	8.8 ± 3.7	6	50.2 ± 12.9	25.6 ± 11.68	48.85%	<0.05	NR	NR	NR	NR
Fluchere et al. [35]	2014	188	61 ± 7	12 ± 4	12	33.6 ± 13.3	13.2 ± 9.1	61.00%	<0.001	1173 ± 495	636 ± 376	46.00%	<0.001
This study	2020	90	57.43 ± 7.85	11.67 ± 4.75	6	38.11 ± 13.96	21.48 ± 12.33	43.60%	<0.001	1448.0 ± 546.93	483.99 ± 330.42	66.60%	<0.001

Data are presented as: mean ± standard deviation UPDRS, Unified Parkinson’s Disease Rating Scale; LEDD, Levodopa equivalent daily dose; NR, Not reported. * On stimulation.

**Table 5:** Patients’ characteristics and clinical measurements in patients who underwent bilateral subthalamic nucleus deep brain stimulation under local and general anesthesia.

	Medication	General Anesthesia (n = 90)	Local Anesthesia (n = 56)	*p* Value
Patient characteristics				
Age		57.43 ± 7.85	58.91 ± 8.65	0.2893
Sex				0.8110
Male		42(46.67%)	25(44.64%)	
Female		48(53.33%)	31(55.36%)	
Symptom duration		11.67 ± 4.75	10.55 ± 4.89	0.1753
Medication duration		9.82 ± 3.89	8.98 ± 3.81	0.2027
Baseline measurement				
Total UPDRS	On	31.86 ± 16.96	32.87 ± 17.76	0.7315
	Off	65.93 ± 20.42	68.53 ± 20.34	0.4569
UPDRS Part III	On	20.83 ± 10.96	21.40 ± 12.90	0.7764
	Off	38.11 ± 13.96	40.42 ± 15.30	0.3521
H & Y	On	2.36 ± 0.63	2.30 ± 0.63	0.6280
	Off	3.05 ± 0.82	3.24 ± 0.91	0.1918
ADL	On	82.89 ± 16.86	80.27 ± 15.45	0.3474
	Off	47.44 ± 23.54	50.00 ± 22.18	0.5154
Dyskinesia Disability		2.72 ± 1.31	2.21 ± 1.39	0.0294 *
LEDD (mg/day)		1448.00 ± 546.93	1031.63 ± 451.08	<0.0001 *
MMSE		27.61 ± 2.52	26.53 ± 2.76	0.0273 *
BDI		17.72 ± 10.28	19.00 ± 10.82	0.4931
SF-36 Physical health		156.25 ± 72.58	132.86 ± 61.09	0.0493 *
SF-36 Mental health		177.62 ± 80.37	150.39 ± 72.59	0.0433 *

* *p* < 0.05.

**Table 6:** Summary of clinical outcomes of bilateral subthalamic nucleus deep brain stimulation under local and general anesthesia.

	Medication	Anesthesia	Baseline	6 Month ^*^	*p* Value ^**^	*p* Value ^***^
Total UPDRS	On	General	31.86 ± 16.96	24.53 ± 14.95	0.004	0.205
		Local	32.87 ± 17.76	29.29 ± 14.19	0.429	
	Off	General	65.93 ± 20.42	40.32 ± 21.42	<0.001	0.120
		Local	68.53 ± 20.34	47.26 ± 17.85	<0.001	
UPDRS Part III	On	General	20.83 ± 10.96	16.20 ± 9.46	0.005	0.696
		Local	21.40 ± 12.90	16.67 ± 9.35	0.063	
	Off	General	38.11 ± 13.96	21.48 ± 12.33	<0.001	0.136
		Local	40.42 ± 15.30	24.68 ± 12.51	<0.001	
H & Y	On	General	2.36 ± 0.63	2.24 ± 0.61	0.238	0.853
		Local	2.3 ± 0.63	2.33 ± 0.68	0.959	
	Off	General	3.05 ± 0.82	2.35 ± 0.61	<0.001	0.053
		Local	3.24 ± 0.91	2.64 ± 0.72	0.002	
ADL	On	General	82.89 ± 16.86	84.80 ± 14.22	0.435	0.592
		Local	80.27 ± 15.45	82.16 ± 15.66	0.247	
	Off	General	47.44 ± 23.54	61.98 ± 25.58	<0.001	0.500
		Local	50 ± 22.18	66.92 ± 18.53	<0.001	
Dyskinesia Disability		General	2.72 ± 1.31	1.04 ± 1.27	<0.001	0.062
		Local	2.21 ± 1.39	0.79 ± 1.21	<0.001	
LEDD (mg/day)		General	1448.00 ± 546.93	483.99 ± 330.42	<0.001	0.000
		Local	1031.63 ± 451.08	461.3 ± 284.65	<0.001	
MMSE		General	27.61 ± 2.52	27.23 ± 2.33	0.314	0.621
		Local	26.53 ± 2.76	25.78 ± 3.71	0.493	
BDI		General	17.72 ± 10.28	16.57 ± 10.56	0.473	0.277
		Local	19 ± 10.82	19.78 ± 9.68	0.524	
SF-36 Physical health		General	156.25 ± 72.58	203.14 ± 90.03	<0.001	0.600
		Local	132.86 ± 61.09	188.34 ± 74.5	<0.001	
SF-36 Mental health		General	177.62 ± 80.37	206.88 ± 84.62	0.021	0.988
		Local	150.39 ± 72.59	181.44 ± 80.95	0.076	

* DBS on, ** between baseline and follow-up, *** between two groups: general and local anesthesia.
